# Approximate Subgraph Matching-Based Literature Mining for Biomedical Events and Relations

**DOI:** 10.1371/journal.pone.0060954

**Published:** 2013-04-17

**Authors:** Haibin Liu, Lawrence Hunter, Vlado Kešelj, Karin Verspoor

**Affiliations:** 1 National Center for Biotechnology Information, Bethesda, Maryland, United States of America; 2 University of Colorado School of Medicine, Aurora, Colorado, United States of America; 3 Faculty of Computer Science, Dalhousie University, Halifax, Nova Scotia, Canada; 4 National ICT Australia, Victoria Research Lab, Melbourne, Victoria, Australia; University of Illinois-Chicago, United States of America

## Abstract

The biomedical text mining community has focused on developing techniques to automatically extract important relations between biological components and semantic events involving genes or proteins from literature. In this paper, we propose a novel approach for mining relations and events in the biomedical literature using approximate subgraph matching. Extraction of such knowledge is performed by searching for an approximate subgraph isomorphism between key contextual dependencies and input sentence graphs. Our approach significantly increases the chance of retrieving relations or events encoded within complex dependency contexts by introducing error tolerance into the graph matching process, while maintaining the extraction precision at a high level. When evaluated on practical tasks, it achieves a 51.12% F-score in extracting nine types of biological events on the GE task of the BioNLP-ST 2011 and an 84.22% F-score in detecting protein-residue associations. The performance is comparable to the reported systems across these tasks, and thus demonstrates the generalizability of our proposed approach.

## Introduction

Systems biology investigates the complex interactions between various components of biological systems, and the consequential impacts of these interactions on the function and behavior of the systems. Text mining of the biomedical literature has been shown to be an effective way of automatically extracting important relations between biological components such as protein-protein interactions (PPI) [1]–[3] and protein-disease associations [4], [5], and semantic events involving genes or proteins including gene expression, binding, or regulatory events [6], [7].

While a relation generally involves a pair of entities with different participating roles, linked by a semantic relation type, an event typically captures the association of multiple participants of varying numbers and with diverse semantic roles [8]. Automatic extraction of such knowledge from literature serves as the basis for a broad variety of applications in systems biology, ranging from the identification of molecular pathways to the automatic enrichment of biological process databases (i.e. biocuration). Relations and events can serve as participants in other events; the extraction of such nested event structures also facilitates the construction of complex conceptual networks.

**Figure 1:**
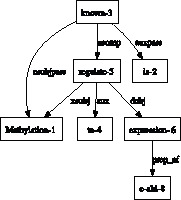
Dependency Graph of “Methylation is known to regulate expression of c-abl.”

Graphs provide a flexible structure to represent a network and naturally describe the interactions between its components. Therefore, they are a powerful primitive for modeling relations and events. In this work, we take advantage of dependency graphs that capture syntactic relations in sentences of natural language text, based on state-of-the-art natural language parsers that can achieve accuracies in the 80–90% range [9]–[11] on parsing biomedical text. Using nodes to represent words in the sentence and edges to decribe governor-dependent relations between words (e.g. Figure 1), dependency graphs can capture long-range dependencies among sentential constituents by considerably narrowing the linear order distance between target entities [12]. Also, the syntactic dependencies closely approximate the underlying semantic relationships [13]. Therefore, they have been effectively used by biomedical knowledge extraction systems [1], [3], [10], [11], [14]. There have been two primary approaches used to integrate dependency graphs with supervised machine learning methods for extracting relational knowledge: feature-based approach and kernel-based.

The feature-based approach encodes node tokens, edge labels and path structures of variable depths of a dependency graph as syntactic features, together with lexical features such as morphological characteristics and bag-of-word frequencies of token texts, to feed learning algorithms [12], [15]. A prominent system adopting this approach is the Turku Event Extraction System (TEES), which has been successfully applied to various relation and event extraction tasks in the biomedical domain [12], [16]–[18]. However, it is often difficult for the feature-based approach to fully capture the rich, structured information represented by a graph without the burden of feature engineering [19], [20]. The kernel-based approach used in conjunction with Support Vector Machines (SVM) is able to use that structure directly. The approach employs a graph kernel that directly calculates the similarity between two dependency graphs. Various graph kernels have been proposed to compare two graphs according to different characteristics of their substructures. The shortest path kernel focuses on the shared information on the shortest dependency path between the constituent entities of a relation [21], the walk-based kernel looks more closely into the shared information inside the shortest path by exploring all possible contiguous subpaths [20], and the all-paths graph kernel considers weighted shared dependency paths of all possible lengths between words [2]. These graph similarity metrics have been applied to extracting protein-protein and drug-drug interactions [2], [3], [22], and biological events [15].

On the other hand, graph matching-based techniques that directly operate on dependency graphs have also proven effective for information extraction tasks in both general English and biomedical domains. A dependency graph matching module was introduced to compute the text relatedness between student answers and correct answers in assisting the automatic grading of student answers [23]. A graph matching approach was also performed on the dependency graphs of two texts to automatically find whether one text is entailed by the other [24]. In addition, given dependency graphs of question and answer sentences, a method was proposed to learn graph-based question answering rules by extracting the maximum common subgraph of two graphs, which determines the common information between a question and an answer sentence [25]. These approaches achieved accuracy figures comparable to state-of-the-art supervised methods.

More recently, we proposed an approach based on exact subgraph matching (ESM) for mining various relations and events from literature in the biomedical domain [26]–[30] (A Java implementation of the ESM algorithm is available at http://esmalgorithm.sourceforge.net). The key contextual structures are learned from each labeled positive instance and maintained as event rules in the form of subgraphs. Event extraction is modeled as a subgraph matching problem by searching for a subgraph isomorphism between rules and input texts. When applied to the GENIA Event (GE) task of the BioNLP-ST 2011 [7], our approach achieves an overall 66.41% precision through the official online evaluation [28]. This precision is superior to all prior published results on the GE task; only three individual systems have achieved a precision in the 60% range. This indicates that the method is effective at precisely identifying events based on the dependency graphs.

However, the overall performance of our ESM-based approach is limited by lower coverage, with an 11% recall deficit contributing to the 7.3% F-score difference with the best individual system. Careful error analysis suggests that the syntactic dependencies encoded in the rules are not sufficient to capture the variety of textual surface forms used to express biological processes. We attribute this problem to the inherent, restrictive property of the exact subgraph matching algorithm that strictly requires that all nodes and connections between nodes in one graph find their injective matches in the other. Although ensuring a high precision, this requirement does not allow partial matching, and therefore limits the generalization potential of the graph representation of rules, leading to the lower recall. In this work, we introduce a novel approach for relation and event extraction based on approximate subgraph matching (ASM). By including a certain degree of error tolerance into the graph matching process, the approach increases the chance of retrieving relational knowledge encoded within complex dependency contexts, while maintaining the extraction precision at a high level. We have successfully applied it in two biological relation/event extraction tasks, achieving results competitive with the state-of-the-art methods, demonstrating the generalizability of our proposed approach.

The rest of the paper is organized as follows: In Section 2, we review recent research advances in mining biological relations and events. Section 3 describes our ASM-based event extraction approach. Section 4 demonstrates two applications in which our approach has been successfully applied. Finally, Section 6 summarizes the paper and introduces future work.

### Related Work

With state-of-the-art protein annotation methods achieving a reasonable 88% F-score [31], the biomedical text mining community has focused on developing techniques to automatically extract from literature important relations between biological components and semantic events involving genes or proteins. Recently, a diversity of extraction methods have been proposed.

Airola *et al.* proposed an all-paths graph (APG) kernel for extracting protein-protein interactions (PPI), in which the kernel function captures weighted shared dependency paths of all possible lengths between words [2]. Thomas *et al.* adopted this kernel as one of the three models used in the ensemble learning for extracting drug-drug interactions [22] and won the recent DDIExtraction 2011 challenge [32]. Qian *et al.* proposed an interesting dependency-motivated constituent tree kernel to extract PPIs. The tree representation generated from a constituent parser is refined using the dependency path information derived from a dependency parser to simplify the tree while retaining its rich, structured information. Their approach achieves the best reported results on the five benchmark PPI corpora.

In addition to binary relations, the BioNLP-ST 2009 shared task included a more ambitious task of detecting complex, nested event structures. It successfully drew interest from 24 teams and has since served as the platform for many studies on event extraction.

The Turku Event Extraction System (TEES) used multi-class SVM classifiers incorporating a wide array of features capturing both linear and dependency contexts to extract arguments of biological events [12]. A linear kernel was adopted with over 430,000 features. The TOKYO system extended the TEES by replacing its manually crafted rule component for combining extracted event triggers and arguments with a machine learning approach, in which rich features collected from classification steps for triggers and arguments are recombined [33]. The JULIE lab adapted the APG kernel to event extraction using syntactically pruned and semantically enriched dependency graphs [15].

BioNLP-ST 2011 extended BioNLP-ST 2009, addressing a wider range of text types, event types, and subject domains. Riedel *et al.* designed the “UMass” system using a discriminatively trained model that jointly predicts trigger tokens, event arguments and protein pairs in binding events [34]. McClosky *et al.* introduced an event extraction system which extended the function of an existing reranking dependency parser [35]. The combination of the underlying models of these two systems achieved the best performance in BioNLP-ST 2011 [36], [37].

As the only rule-based system among the top 5 systems of BioNLP-ST 2009, the “ConcordU” team carefully analyzed 2,000 automatically derived dependency relation paths involved in expressing biological events, and manually coded 27 dependency path patterns which were then applied sequentially to identify event participants [38]. In BioNLP-ST 2011, they extended their work into a two-phase methodology in which the first phase generalizes syntactic dependency relations into a semantic interpretation while the second phase constrains the interpretation with domain-specific knowledge, achieving competitive results [39]. More recently, Bui *et al.* also proposed a rule-based event extraction approach [40]. Training data are first mapped into pre-defined structured representations, from which rules are automatically learned using a list of semantic and linear shallow syntactic features, and further combined to form decision tables for determining event arguments. When evaluated against the GE task of BioNLP-ST 2011, their performance is comparable to the state-of-the-art systems.

As one of the participating teams in BioNLP-ST 2011, we proposed an exact subgraph matching (ESM)-based method for event extraction [27]. The method was then successfully adapted to extract other types of relational knowledge from literature. It achieved an 80% F-score in detecting protein-residue associations [29] in the Nagel corpus [41], and the second best F-score in extracting protein-protein interactions [42] from the largest PPI corpus, BioInfer [43].

An index-based approximate subgraph matching tool [44], SAGA, was previously developed for aligning and querying biological pathways in order to handle the noisy and incomplete characteristics of biological graphs. Their graph matching model considers node gaps, node mismatches and graph structural differences. Haghighi *et al.* [24] also explored an approximate notion of subgraph isomorphism in a textual inference task to measure the semantic overlap between two general English texts via various relaxed graph matching conditions. In this paper, we introduce an approximate subgraph matching (ASM) algorithm designed specifically for literature-based relational knowledge extraction. In contrast to the graphs targeted by SAGA, our algorithm focuses on matching labeled, attributed, directed graphs derived automatically from natural language parsers. Also, the algorithm aims to provide a fine-grained classification on semantic roles of event participants compared to general text entailment tasks. To the best of our knowledge, this is the first attempt to apply approximate graph matching techniques into relational knowledge extraction.

### Relation/Event Extraction Method

In this section, we first introduce the framework of our ASM-based approach. We then describe in detail the core components of the framework in the context of biological event extraction. Next, we formally illustrate our ASM algorithm, and investigate its complexity. Finally, we compare the ASM with existing graph distance/similarity metrics in terms of the different aspects considered in the process of graph comparison.

### ASM-based Event Extraction Framework

Interactions among biological entities are expressed in various ways in the biomedical literature. The underlying assumption of our approach is that the contextual dependencies of each stated biological relation or event represent a typical context for such events in the biomedical literature. Our approach falls into the machine learning category of instance-based reasoning [45]. Specifically, the key contextual structures are learned from each labeled positive instance in a set of training data and maintained as event rules in the form of subgraphs. When compared against unseen text, rules are relaxed in the matching phrase according to different graph matching criteria to identify instances in accordance with rules. When multiple rules are detected to match with the same text, all unique instances are retained since it is often the case that several events are described in a single text.

**Figure 2:**
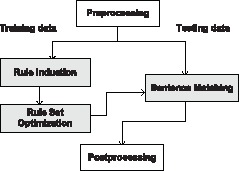
General Architecture of ASM-based Event Extraction.

Figure 2 illustrates the overall architecture of our ASM-based event approach with three core components highlighted: rule induction, sentence matching and rule set optimization. In line with most systems [2], [15], [16], [34], [35], [39], [40], our approach focuses on extracting events expressed within the boundaries of a single sentence. Those that require information across sentences or articles are not considered. It is also assumed that entities involved in the target event have been manually annotated or automatically recognized by upstream procedures.

Several standard preprocessing steps are first completed on both training and testing data. These include sentence segmentation and tokenization, Part-of-Speech (POS) tagging, and syntactic parsing that produces dependency graphs for sentences [46], [47]. Next, we illustrate in detail the core components in the context of biological event extraction.

### Rule Induction

The two BioNLP shared tasks focused on the recognition of biological events from the literature, in a setting where protein mentions are provided in the input [6], [7]. Biological events are characterized in the data by the event type from a predefined set of event types, the trigger that signals the event, and one or more event arguments such as theme or cause of the event which can be a protein or another event.

Event rules are learned automatically using the following method. Starting with the dependency graph of each training sentence, for each annotated event, the shortest dependency path connecting the event trigger to each event argument in the undirected version of the graph is selected. While additional information such as individual words in each sentence (bag-of-words), sequences of words (n-grams) and semantic concepts is typically used in the state-of-the-art supervised learning-based systems to cover a broader context [2], [12], [15], the shortest path between two tokens in the dependency graph is particularly likely to carry the most valuable information about their mutual relationship [21], [38], [42], [48]. In case that there exists more than one shortest path, all of them are considered. For multi-token event triggers, the shortest path connecting every trigger token to each event argument is extracted, and the union of the paths is then computed for each trigger. For regulatory events that take a sub-event as an argument, the shortest path is extracted so as to connect the trigger of the main event to that of the sub-event.

While the dependencies of such paths are used as the graph representation of the event, a detailed description records the participants of the event, their semantic role labels and the associated nodes in the graph. All participating biological entities are replaced with a single tag, e.g. “BIO_Entity”, to ensure generalization of the learned rules. As a result, each annotated event is generalized and transformed into a generic graph-based rule. Algorithm 1 shows the details of the rule induction. The resulting event rules are categorized into different target event types.

**Algorithm 1** Event Rule Induction Algorithm

**Input:** Dependency graph of a training sentence 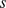, 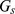; a finite set of annotated biological events that appear in 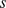, 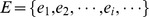, where 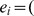 *Type*, *Trigger*, *Arguments
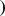
*.

**Output:** A finite set of event rules 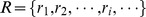.

 1: 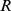 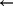 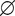


**2:** **for all** 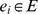 **do**

 3: 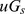 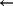 unDirected
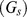


 4: //unDirected() transforms the directed graph 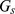 into an undirected graph 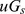


 5: 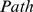 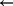 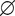//the initial Path set is empty

**6:** **for all** *argument 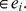
Arguments* **do**

 7: 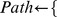 shortestPath
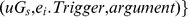


 8: //shortestPath() finds the shortest path(s) between trigger and argument in 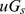


**9:** **for all** *path* 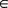 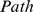 **do**

 10: 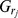 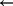 directed
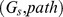


 11: //directed() retrieves the original dependencies of *path* to generate graph representation 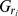


 12: 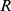 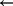 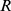 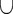 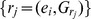


**13: return 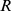
**

For simple events such as *Gene_expression* and *Protein_catabolism* that only involve a trigger and a theme argument, constructing the graph representation for each event is straightforward. However, for complex events such as *Binding* that take varying numbers of proteins as themes, and regulation events that have an optional cause argument in addition to theme, deriving the graph representation deserves more attention. In our previous work, we attempted to compute the dependency path union of all shortest paths from trigger to each event argument, resulting in a graph in which all event participants are jointly depicted [26], [27]. In the event extraction process, this representation is able to identify the trigger and arguments of a complex event simultaneously. On the one hand, since the event participants are considered together for mutual disambiguation, this representation leads to a higher precision in detecting complex events. On the other hand, because the number of complex event arguments varies according to contexts, such graph representation limits its generalization potential. For instance, the graph of a *Positive_regulation* containing both theme and cause arguments cannot be applied to a theme-only event context because of the missing cause argument. Likewise, a graph encoding a *Binding* activity among three proteins cannot identify an event context where only two proteins bind to each other.

In this work, for complex events, in addition to computing dependency path unions, individual dependency paths connecting triggers to each argument are also considered to determine event arguments independently. If the resulting arguments share the same event trigger, they are grouped together to form a potential event. In fact, similar approaches were attempted in both BioNLP shared tasks, and have been proven successful by the best-performing systems [12], [34]. In our approach, the individual paths aim to retrieve more potential events while the path unions retain the advantage of joint learning.

**Figure 3:**
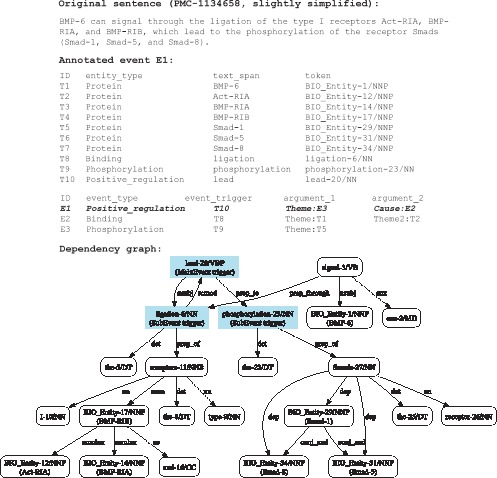
Event Rule Induction Example.

Figure 3 exemplifies the rule induction process for an annotated *Positive_regulation* event of a sentence extracted from an article (PMC-1134658). Labels for proteins and event triggers have been attached to the event annotation. As highlighted in the dependency graph derived by the McClosky-Charniak domain-adapted parser [46], paths that connect triggers of the main event and the sub-events are learned. Since two paths exist between tokens “lead-20/VBP” and “ligation-6/NN”, both are considered in the graph representation, resulting in 5 different event rules, as listed in Table 1. As a path union, the graph encoded in “E1a” or “E1b” subsumes the individual paths represented in other rules.

**Table 1:** Event rule representation.

Rule	Rule Description				Graph
ID	Type	Trigger	Theme	Cause	Representation
E1a	Pos.	lead-20/VBP	Phosphorylation:	Binding:	nsubj(lead-20/VBP, ligation-6/NN)
	reg.		phosphorylation-23/NN	ligation-6/NN	prep_to(lead-20/VBP, phosphorylation-23/NN)
E1b	Pos.	lead-20/VBP	Phosphorylation:	Binding:	rcmod(ligation-6/NN, lead-20/VBP)
	reg.		phosphorylation-23/NN	ligation-6/NN	prep_to(lead-20/VBP, phosphorylation-23/NN)
E1c	Pos.	lead-20/VBP	Phosphorylation:		prep_to(lead-20/VBP, phosphorylation-23/NN)
	reg.		phosphorylation-23/NN		
E1d	Pos.	lead-20/VBP		Binding:	nsubj(lead-20/VBP, ligation-6/NN)
	reg.			ligation-6/NN	
E1e	Pos.	lead-20/VBP		Binding:	rcmod(ligation-6/NN, lead-20/VBP)
	reg.			ligation-6/NN	

### Sentence Matching

Event extraction is achieved by matching the induced rules to each testing sentence and applying the descriptions of rule tokens to the corresponding sentence tokens. Since rules and sentences all possess a graph representation, event recognition becomes a subgraph matching problem. In this work, we introduce a novel *approximate subgraph matching* (ASM) algorithm to identify a subgraph isomorphic to a rule graph within the graph of a testing sentence. The approximate subgraph matching problem in our work is defined as follows.

#### Definition 1

An event rule graph 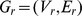 is *approximately isomorphic* to a subgraph of a sentence graph 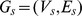, denoted by 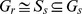, if there is an injective mapping 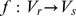 such that, for a given threshold 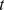, 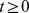, the subgraph distance between 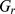 and 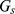 satisfies 0 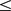 subgraphDist 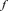
(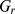, 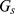) 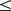 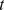, where subgraphDist
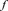
(*G_r_*, *G_s_*) = *w_s_*×structDist
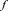
(*G_r_*, *G_s_*)+*w_l_*×labelDist
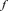
(*G_r_*, *G_s_*)+ *w_d_*×directionality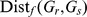, and where the weights 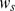, 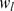, and 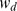, and the distance functions structDist, labelDist, and directionalityDist are defined in the next paragraph.

The subgraph distance computes the cost of transforming a subgraph of the sentence graph into the rule graph, and is proposed to be the weighted summation of three penalty-based measures for a candidate match between the two graphs. The measure **structDist** compares the distance between each pair of matched nodes in one graph to the distance between corresponding nodes in the other graph, and accumulates the structural differences. The distance is defined as the length of the shortest dependency path between two nodes. Because dependency graphs are edge-labeled, oriented graphs, the measures **labelDist** and **directionalityDist** evaluate respectively the overall differences in edge labels and directionalities on the shortest path between each pair of matched nodes in the two graphs. The real numbers 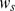, 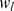 and 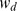 are non-negative weights associated with the measures.

The weights 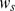, 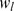 and 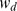 are defaulted to be equal but can be tuned to change the emphasis of the overall distance function. The distance threshold 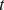 controls the isomorphism quality of the retrieved subgraphs from sentences. A smaller 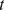 allows only limited variations and always looks for a sentence subgraph as closely isomorphic to the rule graph as possible. It ensures extraction precision by introducing fewer false positive matches, but it may not be able to detect events embedded in more complex contexts. 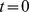 turns the ASM into the exact subgraph matching (ESM) scenario. The ASM thus naturally subsumes the ESM. A larger 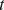 enables the extraction of events described in complicated dependency contexts, thus increasing the chance of retrieving more events. However, it can incur a bigger search cost due to the evaluation of more potential solutions. 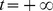 corresponds to a search for the co-occurrence of all rule nodes in a sentence without considering contextual constraints. The formal ASM algorithm and an analysis of its complexity are presented in the next section.

Compared to binary relation extraction tasks, the challenge of event extraction lies in the aim of recognizing complex and nested events. For instance, simple events can serve as arguments of complex events, and complex events themselves may also act as participants of other complex events. Therefore, an iterative, bottom-up matching process is proposed in this work.

**Figure 4:**
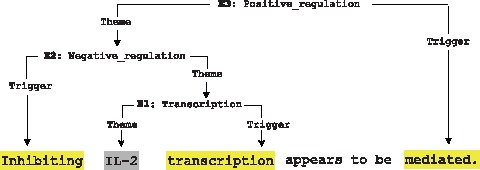
Iterative Bottom-up Event Extraction Example.

Starting with the extraction of simple events, simple event rules are first matched with a testing sentence. Next, as potential arguments of higher level events, obtained simple events continue to participate in the subsequent matching process between complex event rules and the sentence to initiate the iterative process for detecting complex events with nested structures. The process terminates when there is no new candidate event generated for the testing sentence. Figure 4 illustrates a simple example of the bottom-up process to extract three chained events from a sentence (PMID-10229815).

In Section “Rule Induction” we showed that the graph representation of our induced rules, even for complex events, is “simple” in the sense that higher-order constructs are not explicitly encoded in the representation (see Table 1) and indeed the rules are not tied to specific event types. As an example, the theme and the cause of the event rule “E1a” in Table 1 are not restricted to only the annotated types *Phosphorylation* and *Binding* respectively. Rather, such constructs are implicitly captured through references to tokens which are in turn sub-event triggers. We believe that the contextual structures linking annotated sub-events of a certain type are generalizable to other event types. Therefore, during the matching phase we relax the event rules that contain sub-event arguments such that any matched event can substitute for the sub-event. This relaxation increases the chance of extracting complex events with nested structures but still takes advantage of the contextual constraints encoded in the graph representation of rules.

Finally, post-processing is performed to transform raw sentence matching results into the required format according to the event extraction task.

### Rule Set Optimization

Typical of instance-based reasoners, the accuracy of rules with which to compare an unseen sentence is crucial to the success of our approach. As observed in [2], the shortest paths concentrate on the main structure expressing the mutual relationship between nodes and sometimes exclude words that are relevant to specific event contexts. Consequently, although rules are induced from positively labeled events, when the graph representation of a rule is detected in previously unseen text, the encoded contextual dependencies may not always contain a valid event. For instance, a *Transcription* rule encoding a noun compound modification dependency between “TNF” and “mRNA” derived from an event context “expression of TNF mRNA” should not produce a *Transcription* event for the general phrase “level of TNF mRNA” even though they share a matchable dependency. Similarly, “Sp1 transcription” does not express an event but is an adjective to describe “factors” in the context of “Sp1 transcription factors”. Such matches result in false positive events.

Therefore, we measured the accuracy of each rule 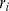 in terms of its prediction result via Eq.(1). Each rule is compared against training sentences using the subgraph matching approach, leaving out the sentence from which the rule was learned. For rules that produce at least one prediction, we ranked them by 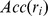 and excluded the ones with a 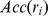 ratio lower than an empirical threshold, e.g. 1∶4. We assume that these rules will produce false positive predictions on unseen text if they are retained in the rule set. Rules that do not make predictions are kept as they may potentially contribute to the testing data.
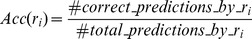
(1)


Because of nested event structures, the removal of some rules might incur a propagating effect on rules relying on them to produce arguments for the extraction of higher order events. Therefore, an iterative rule set optimization process, in which each iteration performs sentence matching, rule ranking and rule removal sequentially, is conducted, leading to a converged, optimized rule set. While the ASM algorithm aims to extract more potential events, this performance-based evaluation component ensures the precision of our event extraction framework.

### Approximate Subgraph Matching Algorithm

The subgraph matching problem is NP-complete [49]. Also, it has been shown that the complexity of the approximate subgraph matching problem is equivalent in complexity to the largest common subgraph problem [50]. However, the graphs of rules and sentences involved in the matching process are small. Therefore, a simple approximate subgraph matching algorithm is feasible in this context. Our ASM algorithm is designed to respect the event rules, since rules are learned from event annotations in which each participant is curated by a domain expert. That is, it searches for a subgraph isomorphism between two graphs by always attempting to transform a subgraph of the sentence graph into the rule graph. The main and essential subroutines of the algorithm are formalized in Algorithms 2 and 3.

The algorithm starts with finding the start nodes for matching. Each rule is allowed to have only one start node while each sentence can possess a set of start nodes. Two scenarios are considered. First, if the rule contains at least one “BIO_Entity” token, the “BIO_Entity” token that has the lowest token number becomes the start node of the rule. This does not reduce the set of found solutions. In the meantime, every “BIO_Entity” token in the sentence becomes an alternate start node for the sentence. Second, if the rule does not have any “BIO_Entity” token, the token with the lowest token number becomes the start node of the rule, while every token in the sentence becomes a candidate start node. The second scenario applies to regulatory event rules that only use sub-events as arguments.

The *for* loop of lines 7–25 attempts to match the rule graph to the sentence graph, starting from matching the rule start node with each sentence start node. Next, potential matching nodes in the sentence are retrieved for each of the remaining rule nodes in order to generate all candidate injective node matches between the two graphs. Each candidate is then evaluated to compute a corresponding subgraph distance.

#### Relaxing node matches

**Figure 5:**
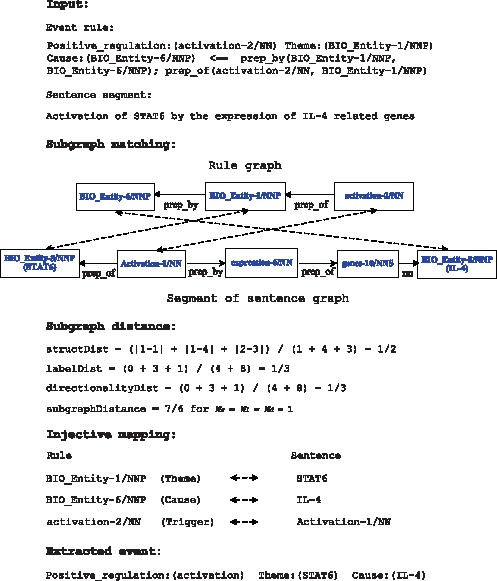
ASM-based Event Extraction.

When comparing two graph nodes in the 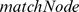 method, various node features can be considered, resulting in different matching criteria. The features include POS tags (P), event trigger (T), token lemmas (L) and tokens (A), ranging from the least specific matching criterion, P, to the much stricter criterion, A. For each sentence, the algorithm returns all the matched rules together with the injective mappings from rule nodes to sentence tokens. Events are then extracted by applying the descriptions of tokens in each matched rule (e.g. role labels) to the corresponding tokens of the sentence. Figure 5 presents a detailed example of the ASM-based event extraction for a *Positive_regulation* event. The matching criteria, “P*+L”, require that the relaxed POS tags (P*) and the lemmatized form (L) of tokens be identical for each rule node to match with a sentence node. The relaxed POS allows the plural form of nouns to match with the singular form, and the conjugations of verbs to match with each other. The BioLemmatizer [51] is used to generate lemmas.

**Algorithm 2** Approximate Subgraph Matching Algorithm (Main algorithm)

**Input:** Dependency graph of a testing sentence 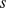, 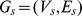 where 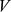 is the set of nodes and 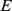 is the set of edges of the graph; a finite set of biological event rules 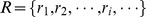, where 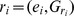. 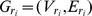 is the dependency graph of 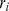; a given subgraph distance threshold 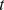


**Output:** 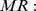 a set of biological event rules from 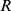 matched with 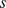 together with the injective mapping

**Main algorithm:**

 1: 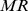 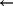 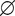


**2:** **for all** 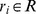 **do**

 3: 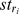 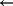 startNode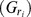//startNode() finds the start node 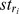 of the rule graph 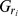


 4: 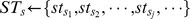//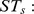 the set of start nodes of the sentence graph 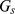


**5:** **for all** 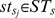 **do**

 6: **if** matchNode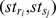 **returns** FALSE **then**

 7: //matchNode() checks if an injective match exists between two nodes

 8: go to Line 5

 9: **else**

 10: 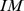 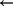 (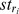, 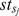)//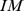: record injective matches between nodes in 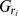 and 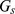


**11** **for all** remaining nodes 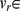 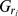 **do**

 12: 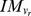 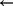 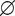//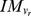: record injective matches between 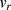 and nodes in 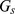


**13:** **for all** remaining nodes 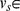 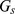 **do**

 14: **if** matchNode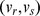 **returns** TRUE **then**

 15: //matchNode() assesses if two nodes can be matched using node features

 16: 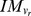 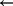 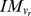 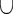 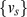


 17: **if** 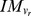 is empty **then**

 18: go to Line 5

 19: 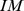 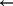 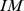 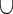 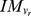


 20: 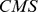 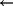 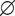 // 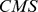: record candidate node matching schemes in 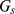


 21: 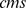 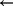 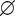 // a candidate node matching scheme

 22: 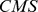 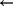 combinMatching(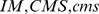)

 23: //combinMatching() recursively generates all candidate node matching schemes in 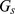


**24:** **for all** candidate matching 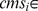 *CMS* **do**

 25: **if** subgraphDistance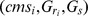 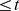 **then**

 26: 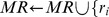 with cms_i}

**27:** **return** 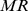


#### ASM algorithm complexity

Let us assume that the sentence graph 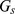 and the rule graph 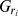 have 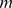 and 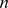 vertices, and 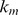 and 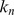 edges respectively. The algorithm complexity is estimated to be 
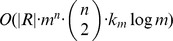
. As we could expect, since the problem of subgraph matching is known to be NP-hard, the complexity is exponential. The main source of inefficiency is the generation of candidate node matching schemes which explores all potential solutions in a sentence, giving 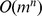 possible schemes. The subgraph distance function is called for each candidate solution, which makes 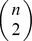 invocations of shortest path calculation for pairwise comparison of dependency contexts of matched nodes in two graphs. Dijkstra’s algorithm [52] requires 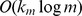 time to compute the shortest path between any two nodes in 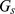.

However, we have observed that the algorithm is relatively efficient in practice and we have successfully run it on several event and relation extraction tasks. We show that this efficient performance in practice can be expected. First, on average there are about 24 words in a sentence in the biomedical text [53], and therefore 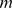 and 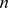 are generally small. Second, since injective matches are required between graphs, the solution space is effectively narrowed down from 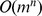. As a result, the algorithm is alleviated by only evaluating a small subset of all possible matches, e.g., 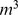 vs. 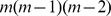. Third, by matching from pairs of start nodes, the number of comparisons is efficiently reduced. In practice, it takes the ASM less than a second to match a total of 13,000 rules of different event types with a sentence and return results.

**Algorithm 3** Approximate Subgraph Matching Algorithm (Subroutines)

**Subroutine:** combinMatching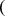 *IM, CMS, cms
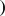
*

 1: 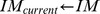; 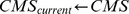; 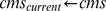


 2: //assign 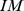, 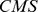 and 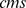 from the parent level to the current 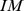, 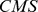 and 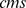


 3: **if** 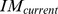 is empty **then**

 4: 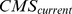 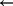 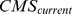 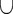 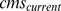


 5: **return** 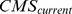


 6: pop 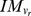 from 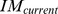


**7:** **for all** 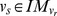 **do**

 8: 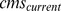 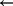 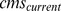 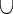 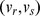


 9: **if** 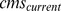 is an injective matching scheme **then**

 10: 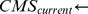 combinMatching(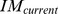, *
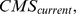
* 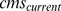)

 11: **return** 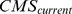


**Subroutine:** subgraphDistance
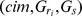


 1: 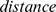 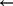 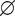


 2: 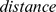 = 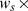 structDist(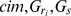)+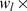 labelDist(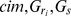)+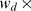 directionalityDist(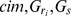)

 3: //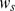, 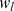 and 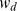 are the weights for each component

 4: **return** 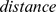


**Subroutine:** structDist(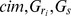)

 1: 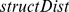 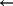 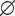; 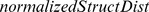 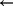 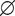


**2: for all** injective matching pairs (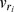, 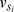) and (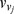, 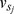) 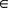 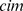 **do**

 3: 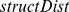
+ = 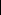 shortestPathLength(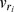, 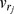, 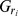) 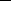 shortestPathLength(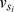, 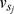, 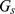) 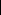


 4: 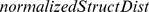 = 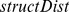/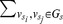 shortestPathLength(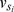, 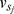, 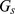)

**5: return** 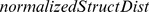


**Subroutine:** labelDist(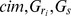)

 1: 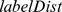 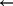 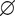; 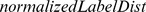 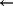 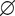


 2: create two empty stacks 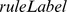 and 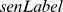


**3: for all** injective matching pairs (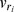, 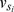) and (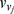, 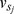) 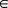 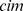 **do**

 4: push Label(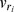, 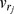, 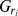) onto 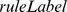


 5: //Label() returns all labels on the shortest path between nodes

 6: push Label(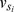, 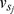, 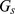) onto 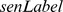


 7: 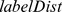
+ = diffLabel(Label(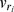, 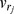, 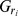), Label(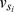, 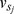, 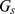))

 8: //diffLabel() returns the number of different labels between two stacks

 9: 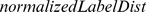 = 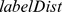/(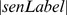
+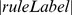)

**10: return** 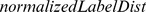


**Subroutine:** directionalityDist(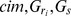)

 1: 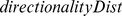 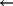 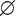; 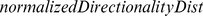 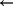 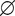


 2: create two empty stacks 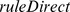 and 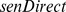


 3: **for all** injective matching pairs (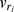, 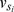) and (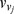, 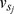) 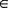 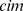 **do**

 4: push Direction(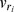, 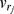, 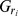) onto 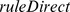


 5: //Direction() returns all directions on the shortest path between nodes

 6: push Direction(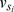, 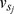, 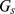) onto 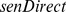


 7: 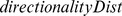
+ = diffDirect(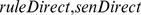)

 8: //diffDirect() returns the number of different directions between two stacks

 9: 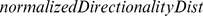 = 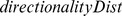/(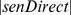
+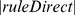)

 10: **return** 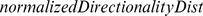


### Comparison with Existing Graph Distance/Similarity Measures

While the cost function of the ASM measures the subgraph distance between two graphs, graph kernels directly compute the similarity between the graphs. Since a distance function can be converted straightforwardly into a similarity measure, we briefly compare the ASM with some existing graph kernel metrics in terms of the different aspects considered in the process of graph comparison.

The edit distance kernel [54] calculates the edit distance between two event dependency graphs, that is, the minimum operations (deletion, insertion and substitution) needed to transform one graph entirely into the other. Since the expression of information about events or relations can be scattered around a sentence, pursuing a global isomorphism requires various operations to deal with instance-specific but event-irrelevant linguistic variation. In the extreme cases, one graph may have to be completely recreated into the other. This explains in part why the edit distance kernel yielded a high precision on all five benchmark PPI corpora (AIMed [55], BioInfer [43], HPRD50 [1], IEPA [56], and LLL [57]) but a significant lower recall compared to other kernel-based methods [3]. Instead of transforming the entire graph, ASM is able to focus only on the event-relevant substructures, and search for a subgraph isomorphism between graphs. Also, the edit distance kernel ignores original edge orientations when transforming graphs into linear path chains to simulate the edit distance calculation between sequences of strings. However, directionality is a crucial indicator of the semantic roles, e.g. agent or patient. The ASM provides a finer classification of event participants by preserving the direction information. Further, the graphs considered in the edit distance kernel are constrained to the shortest dependency path connecting the constituent entities of a relation. While a rule graph is derived from shortest paths, the ASM searches for the corresponding subgraph within a full sentence graph. By exploring a broader context, the resulting subgraph may not correspond to the shortest path connecting targeted entities.

**Figure 6:**
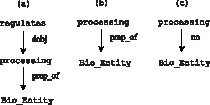
Dependency Kernel Example.

The dependency kernel [20] recursively computes the number of common subgraphs between two dependency graphs. The kernel function relies on the notion of “common child pairs” of node 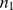 in one graph and node 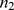 in the other, namely the set of pairs of nodes that have parents 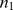 and 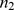 respectively, and that are connected to the parents via the same type of edge. When traversing the graphs in search of common subgraphs, these are the nodes at which the exploration continues. In Figure 6, for instance, 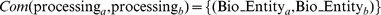 because Bio_Entity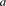 and Bio_Entity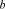 are connected to the parents via the same dependency label “prep_of”. However, due to this restrictive edge match requirement, 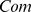
(processing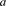, processing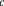) 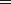
msbm10 at 12 pt 63. Even though “processing of Bio_Entity” is equivalent in meaning to “Bio_Entity processing”, the dependency kernel returns no common structures between graphs 5a and 5c. In contrast, because the ASM models node and edge comparisons independently, it can capture the two common node pairs (processing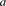, processing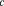) and (Bio_Entity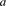, Bio_Entity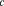), allowing an underlying subgraph isomorphism between 5a and 5c to happen.

The all-paths graph (APG) kernel [2] counts weighted shared paths of all possible lengths between two pairs of candidate entities. The graph representation of the kernel consists of two sub-representations: the full dependency parse and the surface word sequence of the sentence where a pair of interacting entities occurs. At the expense of computational complexity, this representation enables the kernel to explore broader contexts of an interaction, thus taking advantage of the entire dependency graph of the sentence. When comparing two interaction instances, instead of using only the shortest path that might not always provide sufficient syntactic information about relations, the kernel considers paths of all possible lengths between entities. In contrast, the graph representation that the ASM searches in a sentence is inherently restricted to the shortest path between the target entities, as described in Section 3.2. However, although paths of other lengths e.g., the second shortest path can be also obtained to enrich our rule set, one could argue that the performance of the APG may benefit from its word sequence sub-representation because linear, flat feature-based methods have also achieved state-of-the-art results in information extraction (IE) tasks [12], [16], [18]. To the best of our knowledge, while the APG kernel was successfully applied to extract protein-protein and drug-drug interactions [2], [22], no experiments have been conducted to assess the individual contributions of the internal sub-representations of the APG kernel.

In fact, all existing graph kernels are developed to facilitate the extraction of binary relationships, i.e., to help SVM make a decision on whether a co-occurrence of two entities bears a pre-defined relation type. The ASM targets a broader problem definition and is able to identify various components of a relation or event, such as predicate of a relation, and trigger or various themes of an event. However, in order to perform a direct, fair comparison between the ASM and existing graph kernel metrics, the ASM has to also be kernelized. This will allow the ASM to not only take advantage of the capability of SVM that implicitly explores a high dimensional feature space, but also be compared with existing kernels on the same IE tasks. We plan to explore the use of ASM in a graph kernel in future work.

## Applications of ASM-Based Approach

In this section, we evaluate the proposed ASM-based approach on two biomedical applications: BioNLP shared tasks, and Protein-Residue association detection.

### BioNLP Shared Tasks

#### Datasets

We use the dataset of the GENIA Event (GE) task of BioNLP-ST 2011, including training, development and testing sets. This dataset subsumes the BioNLP-ST 2009 dataset of biomedical journal abstracts, but adds full-text articles. Genes and gene products are pre-annotated as “Proteins” and provided in the dataset. The event annotation is only available for training and development sets. Table 2 presents some statistics of the GE dataset. The McClosky-Charniak domain-adapted parser [46], which is among the best performing parsers trained on the GENIA Treebank corpus, is used to parse the sentences. The resulting native constituency output is then transformed to the “collapsed” form of the Stanford dependency scheme [47] to produce dependency graphs using the Stanford parser tools [58]. The parsing results were provided by the shared task organizers [7].

**Table 2:** Statistics of BioNLP-ST 2011 GE dataset (values in parentheses are the numbers of full articles).

Attributes Counted	Training	Development	Testing
Abstracts+Full articles	908 (5)	259 (5)	347 (4)
Sentences	8,759	2,954	3,437
Proteins	11,625	4,690	5,301
Total events	10,287	3,243	4,457
Sentence-based events	9,583	3,058	hidden

#### ASM parameter setting

The GE task includes 9 different event types. Since each type possesses its own event contexts, an individual threshold 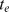 is assigned to each type. Together with the 3 distance function weights 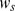, 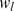 and 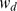, the ASM requires 12 parameters for the event extraction task. Because of the nested event structures, these parameters are correlated and must be tuned simultaneously. Therefore, a genetic algorithm (GA) [52] is used to determine the values automatically using the training data.

Our GA works with a population of potential parameter settings. The values of parameters are encoded by integer values within a predefined range: [0, 50]. For each potential setting, the fitness function of GA performs sentence matching between rules learned from the training set and sentences of the development set, evaluates the corresponding event extraction performance on the development set using the provided gold event annotation, and returns the resulting F-score. GA iterates the fitness function with a goal of maximizing the F-score on the development data.

Our GA is set up to evolve for 50 generations, each of which consists of a population of 100 potential parameter settings. GA starts with a randomly generated population of 100 potential solutions and proceeds until 50 generations are reached. The number of generations and the population size are decided with consideration of the runtime cost of evaluating the fitness function. A large number of generations or population size would incur an expensive runtime cost of evaluation. Table 3 shows the resulting optimized parameter setting with the equal weights 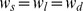 constraint.

**Table 3:** ASM parameter setting for the GE task.

Parameter	Value	Parameter	Value
*t_Gene_expression_*	7	*t_Regulation_*	3
*t_Transcription_*	5	*t_Positive_regulation_*	3
*t_Protein_catabolism_*	7	*t_Negative_regulation_*	3
*t_Phosphorylation_*	10	*w_s_*	10
*t_Localization_*	10	*w_l_*	10
*t_Binding_*	7	*w_d_*	10

#### Event extraction results

Following the proposed framework, rules are first induced from both training and development sets. The resulting rule set is then optimized and matched with the testing sentences using the ASM with the above parameter setting and node matching criteria “P*+L”. The graph-based rules are distributed over the nine event types shown in Table 4. Our performance on the testing set of the GE task is reported in Table 5, evaluated by the primary metric of the task via the official online evaluation (http://bionlp-st.dbcls.jp/GE/eval-test/).

**Table 4:** Distribution of event rules.

Event type	No. of event rules
Gene_expression	2,438
Transcription	479
Protein_catabolism	130
Phosphorylation	282
Localization	281
Binding	1,651
Regulation	1,487
Positive_regulation	4,626
Negative_regulation	1,619
TOTAL	12,993

**Table 5:** GE results on testing set evaluated by “Approximate Span/Approximate Recursive Matching.”

Event type(No. of events)	Recall(%)	Precision(%)	F-score(%)
Gene_expression (1002)	68.66	85.36	76.11
Transcription (174)	47.13	76.64	58.36
Protein_catabolism (15)	53.33	100.00	69.57
Phosphorylation (185)	80.00	71.15	75.32
Localization (191)	45.55	75.65	56.86
[SVT-TOTAL] (1567)	64.65	81.43	72.07
Binding (491)	35.44	54.55	42.96
[EVT-TOTAL] (2058)	57.68	75.94	65.56
Regulation (385)	22.34	42.16	29.20
Positive_regulation (1443)	33.75	54.66	41.73
Negative_regulation (571)	28.55	39.95	33.30
[REG-TOTAL] (2399)	30.68	48.97	37.72
[ALL-TOTAL] (4457)	43.15	62.72	51.12

Table 6 presents the performance comparison results between the ASM and the top individual systems in the BioNLP-ST 2011 that achieved an F-score higher than 50%. Even though the shared task organizers provided participants with uniform supporting analyses [7] on the datasets such as tokenization, POS tagging and syntactic parsing, participating systems may have undergone different preprocessing steps. Also, individual systems are always parameterized and optimized differently. Thus, the performance differences among systems may not fully reflect their underlying methodologies. Rather than a method-level comparison, we intend to show a system-level comparison in which complete systems take the same dataset and produce a solution evaluated against the annotations of the held-out data through a public platform. A run of the ASM with 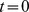 is also listed to show the performance when it corresponds to exact subgraph matching (ESM). The impact of the ASM method itself is reflected through comparison of the optimized ASM with the ASM (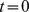), where the same preprocessing was used. In addition to the overall recall, a detailed comparison is also provided for simple events (“SVT”) that only involve a trigger and a theme, *Binding* events (“BIND”) that can take multiple participants of varying numbers, and regulatory events (“REG”) containing diverse semantic roles. Moreover, since the GE task dataset of the BioNLP-ST 2011 subsumes the dataset of BioNLP-ST 2009, we present separately in Table 7 the performance comparison on the BioNLP-ST 2009 data.

**Table 6:** Performance comparison with other systems on the GE task of BioNLP-ST 2011.

System	SVT	BIND	REG	TOTAL		
	F-score	F-score	F-score	Recall	Precision	F-score
UMass [34]	73.50	48.79	43.82	48.49	64.08	55.20
UTurku [16]	72.11	43.28	42.72	49.56	57.65	53.30
MSR-NLP [59]	71.54	41.39	40.02	48.64	54.71	51.50
**ASM**	72.07	42.96	37.72	43.15	62.72	51.12
ConcordU [60]	70.52	36.88	40.16	43.55	59.58	50.32
UWMadison [61]	68.70	36.88	40.37	42.56	61.21	50.21
Stanford [62]	70.88	44.34	35.21	42.36	61.08	50.03
**ASM** ( 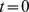 )	68.47	36.21	36.01	37.45	66.41	47.89

**Table 7:** Performance comparison with other systems on the dataset of BioNLP-ST 2009.

System	SVT	BIND	REG	TOTAL		
	F-score	F-score	F-score	Recall	Precision	F-score
UMass [34]	71.54	50.76	45.51	48.74	65.94	56.05
UTurku [16]	70.36	47.50	44.30	50.06	59.48	54.37
MSR-NLP [59]	70.08	43.86	40.85	48.52	56.47	52.20
**ASM**	70.07	43.21	38.78	42.80	64.73	51.53
Stanford [62]	69.29	47.57	36.09	42.55	62.69	50.69
UWMadison [61]	65.13	43.21	41.08	42.17	62.30	50.30
ConcordU [60]	67.75	37.41	40.96	43.09	60.37	50.28
**ASM ( 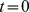 )**	64.78	41.55	36.68	36.77	68.86	47.94

Our approximate subgraph matching-based method achieves an overall 51.12% F-score on the GE task testing data, including both abstracts and full-text papers. Considering that “MSR-NLP” [59] based their work on “UTurku”’s system [16], our performance is comparable to the top systems, and is within a reasonable margin from the best-performing system “UMass”. Our method shows an overall superior precision over most participating teams, of which only three individual systems obtained a precision in the 60% range. Particularly, in the exact subgraph matching scenario (ASM with 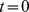), the best precision can be achieved with a constrained recall. The precision of simple events is approaching 89% (88.98%), nearly 8% higher than that of “UMass” (81.40%). This indicates that event rules automatically learned and optimized over training data generalize well to the unseen text. Whenever the graph representation of a rule is detected in testing data, the rule has the ability to identify precisely a corresponding event. Considering that the precision outperforms the “ConcordU” system relying on manually developed patterns [60], it indicates that learned rules can be even more accurate than human-coded rules.

Compared with the exact subgraph matching scenario, the ASM results in a nearly 6% recall gain but still maintains precision at the high level, leading to an important 3.2% increase for F-score. However, a recall deficit of about 5% between the ASM and the top two systems is still observed. Careful error analysis reveals that the difference comes primarily from the extraction of complex events. Specifically, only 23% of the cause arguments for regulatory events that contain both theme and cause (as in Figure 5) are retrieved.

We attributed the missed event arguments to two main reasons. First, information on the shortest dependency path represented in rules is accurate to infer mutual relationship between tokens but sometimes not sufficient to cover all possible linguistic contexts of multi-participant events. Due to missing the relevant event components, even though the ASM attempts to maximize the generalization potential of rules, the corresponding events cannot be identified. As a result, the compound effect of one missing theme of a three-theme *Binding* event will leave the entire event undiscovered, and one missing cause of a regulatory event may incur a chain of unidentified regulation events with nested structures. In contrast, the “UTurku” system employed over 430,000 features including linear surface information, and shallow and deep syntactic structures to capture comprehensive event contexts. “UMass” also attempted to artificially increase the training data size in the shared task in order to obtain more distinguishing features. Our approach simply uses more limited features and hence is not as robust to this linguistic variation.

Second, the current implementation of the injective mapping requirement of the ASM algorithm constrains further generalization of rules. Currently, “P*+L” is used as the matching criteria requiring that the relaxed POS tags and the lemmatized form of tokens be identical when comparing non-“BIO_Entity” nodes in the two graphs. “P*” provides shallow syntactic information but would be too general if used as a standalone criterion. “L” is added to provide specificity. However, although somewhat abstracted from original surface tokens, lemmas are constrained to match at the word level. For further relaxation of node matching, ontology-based, concept-level generalization is necessary. For instance, when “lysine” appears as a rule node, the ASM could allow all amino acids to match with it instead of only looking for this specific residue.

One way to improve the recall of ASM is to provide it with more training data. This can potentially be accomplished through the use of the *distant supervision* paradigm, which automatically creates training instances by heuristically matching existing knowledge to some corresponding text [63]. Next, we demonstrate via an application that combining our ASM-based relation extraction approach with the distant supervision paradigm leads to a state-of-the-art performance.

#### Statistical significance test

While Table 6 demonstrates the important performance differences between the optimized ASM and the ASM (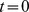) (ESM), in order to claim the contribution of the ASM method itself over ESM we further investigated whether these differences are statistically significant. Since the ASM and the ASM (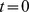) underwent the same preprocessing steps, and were trained and tested on the same datasets, both methods are expected to produce some positively correlated results [64]. Instead of tests that assume independent results from compared methods e.g., the Chi-square test, or a normal distribution on tested samples e.g., paired student’s *t* test, we used the Wilcoxon signed rank test [65], a nonparametric test assuming that there is information in the magnitudes and signs of the differences between paired observations.

Since the gold event annotation of the GE task testing data is hidden to the public, our statistical test is performed on the development data. The 259 documents are randomly divided into 10 groups with 26 documents in 9 groups and 25 documents in the last. Each group is evaluated independently by both optimized ASM and ASM (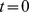), and the score distributions for the two resulting samples are confirmed to be non-Gaussian distributions via the Shapiro-Wilk normality test [65]. The paired samples are then tested by the Wilcoxon signed rank test with the null hypothesis that there is no performance difference between the two methods. Table 8 presents the test results for Precision, Recall and F-score respectively when the level of significance is 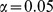.

**Table 8:** Wilcoxon signed rank test results.

WilcoxonTest	ASM	ASM	ASM( 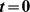 )	ASM( 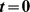 )	*P* value
	*mean*	*std. dev.*	*mean*	*std. dev.*	
Recall	42.26	5.71	36.62	5.52	0.002
Precision	69.39	5.19	72.93	9.14	0.037
F-score	52.40	5.52	48.51	5.70	0.002

The test confirms that the recall and F-score increases from the ASM method itself are statistically significant, as evidenced by the 0.002 *P* value. While according to the test the precision drop of the ASM is also significant (*P* value = 0.037), considering that the change of the balanced F-score is significant, the recall gain provides a more important influence to the overall performance. In spite of the compromise of lower precision for this recall gain, the ASM still achieves a precision higher than most of the reported systems as shown in Table 6 and Table 7. Therefore, we conclude that the ASM significantly increases the chance of retrieving events encoded within complex dependency contexts by introducing error tolerance into the graph matching process, while maintaining the extraction precision at a high level.

### Protein-residue Association

In three-dimensional protein structures, the appearance of certain amino acid residues at key structural positions plays a central role in protein function, for instance enabling ligand or substrate binding. For proteins of therapeutic importance, identifying these protein residues as potential targets is a key early step in drug design. Text mining has been shown to play an important role in such protein function prediction [66]. In this work, we applied our approach to extract protein-residue associations in the biomedical literature.

#### Dataset

**Figure 7:**
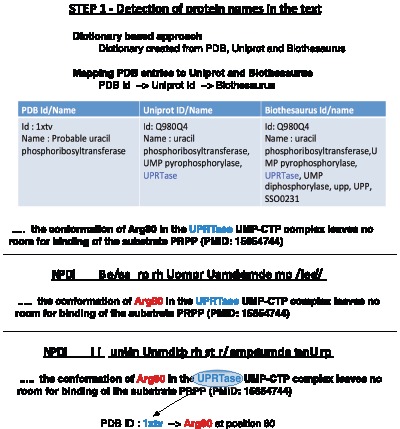
Physical Validation of Protein Residue Relation.

Instead of manually curated annotations, sentences that contain high confidence protein-residue relationships are prepared via distant supervision using Protein Data Bank (PDB) as the biological knowledge source to drive relation extraction learning. Sentences in which at least one protein and one amino acid co-occur are selected from 18,045 abstracts of the primary references for the PDB entries. These sentences are further filtered to retain only those that contain physically validated relationships, i.e., the protein-residue co-occurrence can be substantiated by a physical match of the particular residue to the mentioned protein according to its PDB record (see [66] for more details). While a dictionary lookup is performed to pre-annotate protein names, linguistics-based patterns are used to identify residue mentions and the particular position where they occur in protein sequences [29]. As exemplified in Figure 7, for the sentence “CTP binding affects the conformation of Arg80, and the Arg80 conformation in the UPRTase-UMP-CTP complex leaves no room for binding of the substrate PRPP. ”, the protein-residue pair (UPRTase-Arg80) is validated via the PDB entry “1xtv”, with PMID-15654744 as the primary citation.

Table 9 shows some statistics of the protein-residue association dataset we built through distant supervision. More details about the construction of the dataset can be found in [29].

**Table 9:** Statistics of Protein-Residue relation dataset.

Attributes Counted	No. of instances
Total abstracts	18,045
Total sentences	138,790
Sentences with co-mentions of protein and residue	5,256
Physically validated protein-residue relations	2,814

#### Relation extraction results

Association rules are induced from sentences for 2,216 physically validated relationships by extracting the shortest paths connecting association arguments. The rule set optimization process involves only one iteration as the task does not contain relations with nested structures. An empirical parameter setting for the ASM is used throughout our experiments in which the three distance function weights are 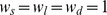 and the single distance threshold 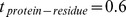.

When evaluated against the remaining 598 physically validated relationships, the ASM with the above parameter setting achieved an 84.22% F-score in extracting protein-residue associations, with an 86.62% recall and an 81.96% precision. The system surpasses a co-occurrence baseline method that assumes a relation when one protein and an amino acid are mentioned together in texts, and a run of the ASM with 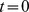, which is equivalent in performance to the exact subgraph matching (ESM) method previously adapted to the protein-residue association problem [29]. Table 10 shows the detailed performance comparison on the constructed dataset. With minor compromise to precision, the ASM brings in a substantial 8% recall increase over ASM (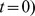, leading to an overall 3.3% F-score improvement.

**Table 10:** Performance comparison on Protein-Residue association extraction.

System	Recall(%)	Precision(%)	F-score(%)
Co-occurrence baseline	100.00	62.42	76.86
**ASM** ( 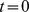 )	78.43	83.60	80.93
**ASM**	86.62	81.96	84.22

Distant supervision helps to relax the reliance of rule induction on curated annotations. Taking advantage of a much broader set of training instances, more rules are reliably learned to cover diverse relation contexts, thus improving the overall coverage of our approach. While distant supervision has been shown effective for system development in relation extraction in the general English domain [67], [68], our work provides additional confirmation of the effectiveness of this strategy in the biomedical domain when integrated with our ASM-based framework.

### Conclusion

In this paper, we proposed a novel approximate subgraph matching-based approach for extracting relational knowledge from biomedical literature. By introducing a certain degree of error tolerance into the graph matching process, our approach increases the chance of retrieving relations or events encoded within complex dependency contexts, while maintaining the extraction precision at a high level. Our approach has been successfully applied to two relation and event extraction tasks. We report results of 51.12% F-score in extracting nine types of biological events of the BioNLP-ST 2011 task and 84.22% F-score in detecting protein-residue associations, demonstrating the generalizability of our approach. In addition, we investigated the complexity of the proposed algorithm, and compared it with existing related graph distance/similarity metrics.

Our approach has a number of advantageous features. First, characterized by high precision, our approach is a preferable choice when accurate information about biological processes is emphasized. It works particularly well on extracting binary relations (including events containing only two participants) with training data where biological entities of the target relation are pre-annotated. Second, although already possessing a reasonable recall, the coverage of the approach can be further increased by integrating distant supervision. Meanwhile, rules learned from co-mentions of pairs of entities known to interact are not prone to over-fitting to an annotated training corpus, thus they are more generalizable across different datasets [42]. This is in contrast with the observation that most state-of-the-art machine learning methods for relation extraction show large performance differences depending on whether or not the evaluation and training instances are taken from the same corpus [3]. Third, our approach is easily adapted to different relation extraction tasks. Its generalizability has been demonstrated via two biomedical applications with various requirements and diverse contexts. The task-specific adaptation only involves specifying the type of the targeted relation, e.g. protein-residue association and retuning the corresponding ASM parameters, and is therefore trivial. Fourth, analyzing extraction errors of the approach is more straightforward compared to SVM-based supervised learning methods as a wrong match can be pinpointed to the specific rule producing it and then corrected.

In our future work, we are interested in extending the proposed subgraph matching algorithm into a graph kernel to be integrated into SVM so that we can take advantage of the capability of state-of-the-art supervised learning methods and compare straightforwardly with existing graph kernel metrics on common information extraction tasks. We would also like to explore some alternative, linguistically-based, methods to relax the current labelDist measure. Currently, a simple strategy in the labelDist measure in the ASM subgraph distance function is used that tracks all different edge labels on the compared paths in two graphs. For instance, even though “prep_of(increase, immunoreactivity) ” in rule possesses the same meaning as “prep_in(increase, immunoreactivity)” in sentence, because “prep_of” is different from “prep_in” in form, labelDist will record a difference of two labels, resulting in a larger labelDist score. Some approaches have been developed to prune or collapse dependency graphs by unifying labels that are equivalent in meaning in order to simplify the graphs [13], [15], [42], [59], [69] which should be applicable here. Finally, we intend to incorporate existing ontologies into the graph matching process to investigate their impact on event extraction performance.
